# Relationship between Health-Related Physical Fitness Parameters and Functional Movement Screening Scores Acquired from a Three-Dimensional Markerless Motion Capture System

**DOI:** 10.3390/ijerph19084551

**Published:** 2022-04-09

**Authors:** Dimitrije Cabarkapa, Joseph M. Whetstone, Aaron M. Patterson, Eric M. Mosier, Damjana V. Cabarkapa, Andrew C. Fry

**Affiliations:** 1 Jayhawk Athletic Performance Laboratory—Wu Tsai Human Performance Alliance, Department of Health, Sport and Exercise Sciences, University of Kansas, Lawrence, KS 66045, USA; d927c184@ku.edu (D.V.C.); acfry@ku.edu (A.C.F.); 2 Sano Orthopedics, 2861 NE Independence Avenue, Lee’s Summit, MO 64064, USA; jwhetstone@sanoorthopedics.com (J.M.W.); apatterson@sanoorthopedics.com (A.M.P.); 3 School of Health Science and Wellness, Northwest Missouri State University, Maryville, MO 64468, USA; emosier@nwmissouri.edu

**Keywords:** aerobic capacity, body fat percentage, body mass index, functionality, vulnerability, explosiveness, anthropometrics, dysfunction, cholesterol

## Abstract

The purpose of the present study was to examine the relationship between five algorithm-derived functional movement screening scores (i.e., readiness, explosiveness, functionality, dysfunction, and vulnerability) obtained from an innovative three-dimensional markerless motion capture system (3D-MCS) and some of the key health-related physical fitness parameters such as maximal aerobic capacity (VO_2_max), body mass index (BMI), body fat percentage (BF%), waist and hip circumferences (WC and HC), and high-density lipoprotein cholesterol (HDL-C). BF% showed a weak positive correlation with vulnerability and moderate-to-strong negative correlations with readiness, explosiveness, and functionality scores. Similarly, but opposite to BF%, VO_2_max showed a weak negative correlation with vulnerability and moderate-to-strong positive correlations with readiness, explosiveness, and functionality scores. BMI, WC, and HC showed moderate negative correlations with vulnerability, readiness, and functionality scores, while HDL-C showed a weak positive correlation with vulnerability and a weak negative correlation with explosiveness scores. Therefore, it appears that 3D-MCS may be used a as a non-invasive testing alternative or in conjunction with currently implemented traditional testing modalities to provide health practitioners with additional information regarding some of the key health-related physical fitness parameters, especially within non-academic environments such as wellness and clinical settings.

## 1. Introduction

A wide range of screening tests have been routinely implemented in clinical settings as precautionary measures for detecting potential issues that may put an individual at an increased risk for developing a certain disease before actual symptoms or signs appear. Being able to quantify the risk for developing certain health conditions early, when they are more easily treatable, is of critical importance for preventive health care and assuring a longer lifespan. Additionally, routine screenings may allow medical practitioners to track an individual’s progress over time and observe changes that may influence the type of intervention that is being prescribed. 

Some of the key health-related physical fitness parameters commonly assessed in clinical settings include body mass index (BMI), waist and hip circumferences, body fat percentage (BF%), and maximal aerobic capacity (VO_2_max) [1]. It has been shown that the previously mentioned parameters are strongly corelated with overall health status, ability to perform daily activities with vigor, and lower prevalence of chronic diseases and their risk factors [1,2]. In addition, previous research has documented a strong inverse relationship between high-density lipoprotein cholesterol (HDL-C) and incidence of coronary heart disease [3,4,5]. Thus, due to its cardioprotective effect, HDL-C has been widely used as one of the key tests for cardiovascular disease risk stratification. 

Alongside health-related physical fitness parameters, functional movement screening has been used for the assessment of musculoskeletal injury risk by identifying limitations or asymmetries in human movement patterns [6,7]. This screening methodology, composed of seven fundamental movement patterns (e.g., deep squat, hurdle step, inline lunge), uses a primitive grading system (e.g., 0–3 scale) and minimal amount of testing equipment to quantify weaknesses and detect areas for improvement. It is primarily devoted to examining the efficacy rather than the ability to perform multiple repetitions of the movement [6,7,8]. However, one of the main issues with this type of screening tool is that assigning various outcome measures for movement quality becomes very subjective. This issue of subjective interpretation of movement quality may be of greater importance especially when dealing with a large cohort of individuals, either in athletic or clinical settings. 

Previous research has been primarily directed towards examining the relationship between traditional functional movement screening scores and musculoskeletal injury risk in athletic settings [9,10,11,12]. Chorba et al. [9] found that the risk for lower body injury was notably greater for collegiate female athletes with functional movement screening scores ≤ 14, which was also identified as a threshold for an increased likelihood of injury within a cohort of professional football players [10]. However, there is a lack of scientific literature focused on examining the relationship between health-related physical fitness parameters and functional movement screenings scores. In one of the recently published studies, Farrell et al. [8] found that functional movement screening scores were significantly associated with waist circumference, BMI, and HDL-C levels. 

Despite exponential technological advancements having occurred, the traditional functional movement screening protocol (i.e., 7 motions, 21 points), which is prone to subjective error measurement, is still being used as a standardized testing modality. Over the last decade, the use of an innovative three-dimensional markerless motion capture system (3D-MCS) has been shown to be a viable method for assessing various types of human motion [13,14,15,16,17], which may serve as an alternative method for objective-centered functional movement screening. This method of assessment could allow practitioners to incorporate a greater number of elementary functional body movements into standardized testing protocols and obtain more information regarding their biomechanical characteristics. Therefore, the purpose of this study was to examine the relationship between health-related fitness parameters and functional movement screening scores acquired from an innovative 3D-MCS. 

## 2. Materials and Methods

### 2.1. Participants

Cross-sectional analyses were performed on data derived from the results of fitness screening tests of apparently healthy individuals (*n* = 307; 219 men and 88 women). All testing procedures performed in this study were approved by the Institutional Review Board.

### 2.2. Anthropometric Measurements

Participant height, measured to the nearest tenth of a centimeter, was assessed using a Harpenden stadiometer (Holtain Limited, London, UK). Body weight, measured to the nearest tenth of a kilogram, was determined as a part of the BF% measurement using the Bod Pod system (Cosmed, The Metabolic Company, Rome, Italy). Participant weight was measured with participants wearing a minimal amount of clothing (i.e., spandex shorts for males and spandex shorts and top for females). BMI was calculated from the measured weight and height (kg·m^−2^). Waist and hip circumferences were measured to the nearest tenth of a centimeter by using a retractable cloth tape measure (Perfect Measuring Tape Company, Toledo, OH, USA) at the top of the iliac crest and at the largest circumference around the buttocks, respectively. 

### 2.3. Body Composition Analysis

BF% was determined using the Bod Pod system (Cosmed, Metabolic Company, Rome, Italy), an egg-shaped air displacement plethysmograph designed to accommodate a wide variety of body shapes and sizes. This system measured the participant’s air displacement to determine the total body volume. Body volume as well as the participant’s previously determined body weight were used to calculate body density and BF%. Participants were tested in the morning following a 12 h fast and were instructed to abstain from exercise two hours prior to the testing. All measurements were obtained with a minimal amount of clothing (i.e., spandex shorts for males and spandex shorts and top for females).

### 2.4. Cardiorespiratory Fitness

The Bruce treadmill testing protocol was used to measure VO_2_max as an indicator of cardiorespiratory fitness. Previous research has shown that the age-adjusted correlations between predicted and measured VO_2_max for this testing modality were strong for both male (*r* = 0.92, SEE = 1.9 mL·kg^−1^·min^−1^) and female (*r* = 0.92, SEE = 2.2 mL·kg^−1^·min^−1^) participants [18,19,20]. Following a warm-up, participants exercised to a volitional endpoint or until specific test-termination-related criteria were achieved (i.e., 85% of age-predicted maximal heart rate and perceived exertion of at least 16 on a 20-point Borg rating of perceived exertion scale). Heart rate during exercise was recorded via electrocardiograph. The time to reach maximal volitional effort while performing the treadmill test was used to predict VO_2_max according to Bruce et al. [18]. In addition, using predicted VO_2_max, age- and sex-specific cardiorespiratory fitness percentiles were identified using maximal treadmill test data derived at Cooper Clinic (Dallas, TX, USA). 

### 2.5. Functional Movement Screening

The functional movement screening protocol used in the present study consisted of 19 body movements performed in sequential order. A detailed description of each movement incorporated into the screening protocol is presented in Table 1. A 3D-MCS system (DARI Motion, Overland Park, KS, USA) composed of eight high-definition cameras recording at 60 fps was used to obtain biomechanical parameters (i.e., kinetics and kinematics) of each body motion. The cameras were positioned at different orientations to surround and cover the testing or screening area. The visual hull technology model records and subtracts the visual signal minus the background, which is being used to generate a pixelated person to obtain biomechanical parameters of each body movement. Prior to each testing session, the system calibration was performed following manufacturer recommendations. For the purpose of consistency, a trained research team member demonstrated how each movement was supposed to be performed while facing the participant and standing outside the camera capture area. Following the instruction or demonstration of each individual movement, the participant received the following command “one, two, three, begin”. At the command “begin”, the participant started the specific movement while the motion was simultaneously being recorded by a 3D-MCS. At the completion of the movement, the research team member gave the command “done” as a signal for the second member in the research team to stop the motion-related recording. The instructions for completion of all 19 body movements remained identical across all participants.

After the completion of the functional movement screening protocol, the data were automatically processed (~30 s) by DARI Motion-defined algorithms to derive the following five unique scoring scales or variables: readiness, explosiveness, functionality, dysfunction, and vulnerability. The readiness scale is a cumulative score based on the overall performance capture analysis and is defined as an explosiveness scale plus a functionality scale minus a dysfunction scale. The explosiveness scale consists of data related to jump heights and is an aggregate of all jump performances. The functionality scale is the accumulation of squat depth captures and represents an aggregate of all squat-related motions. The dysfunction scale attempts to capture and combine motion-related asymmetries (e.g., lower-limb and trunk asymmetries), as well as knee valgus, lower-limb kinetic chaining, and balance-related performances. The vulnerability scale is the aggregate of the following motion capture characteristics: overall performance, stress-related captures (e.g., unilateral high forces, joint flexions, joint torques), and compensation patterns (e.g., overuse of dominant side, limited usage due to history of injury). The vulnerability scale is presented as a percentage (0–100% range), while the rest of the scales are represented in arbitrary units (a.u.). 

### 2.6. High-Density Lipoprotein Cholesterol

Based on the previous research, some speculation has been raised suggesting that HDL-C may be positively associated with lean muscle mass [8]. To further explore this hypothesis, as a secondary, pilot-focused aim of the study, the HDL-C value (mg·dL^−1^) derived from the 12 h fasting lipid analysis of each participant was included as a health-related physical fitness screening parameter.

### 2.7. Statistical Analysis

Descriptive statistics, means and standard deviations ($\bar{x}$ ± SD), were calculated for each dependent variable. T-tests with unequal variance for continuous variables were used to test for significant differences between males and female participants. Pearson product–moment correlation coefficients (*r*) were used to inspect relationships between functional movement screening scores and health-related fitness parameters of interest. Linear regression analysis was used to estimate unstandardized coefficients and their contribution to the prediction model. Statistical significance was set a priori to *p* < 0.05. All statistical analyses were completed with SPSS (Version 26.0; IBM Corp., Armonk, NY, USA) and Microsoft Excel (Microsoft Corp., Redmond, WA, USA). 

## 3. Results

Demographic data and health-related physical fitness test results are presented in Table 2. Of the 307 participants, 219 (71%) were males and 88 (29%) females. The age difference between male and female participants was not statistically significant (*p* = 0.990). While no difference in hip circumference was observed between male and female participants (*p* = 0.431), height (*p* < 0.001), weight (*p* < 0.001), and waist circumference (*p* < 0.001) values were notably greater for males. Despite BMI being only somewhat greater for males when compared to females, this difference was statistically significant (*p* < 0.001). The BF% was significantly greater for the female participants compared to the males (*p* < 0.001). Predicted VO_2_max was significantly greater for male participants (*p* < 0.001). However, percent fitness, which is sex-specific in derivation, was not significantly different between the sexes (*p* = 0.310). HDL-C was significantly greater (*p* < 0.001) for females compared to males. Mean values for the readiness (*p* < 0.001), explosiveness (*p *< 0.001), functionality (*p* < 0.001), and dysfunction (*p* = 0.010) scores, although only somewhat greater for men compared to women, were significantly different between sexes. The mean vulnerability score was slightly greater for females when compared to males, and this difference was also significant (*p* < 0.001). 

**Figure 1:**
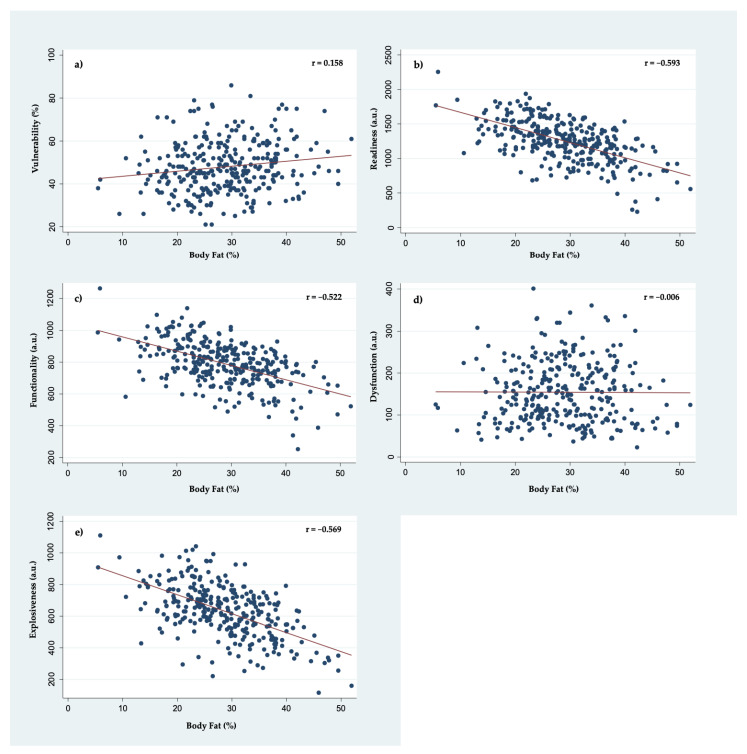
Correlation plots of BF% with (**a**) vulnerability, (**b**) readiness, (**c**) functionality, (**d**) dysfunction, and (**e**) explosiveness scores derived from a 3D-MCS during functional movement screening protocol.

**Figure 2:**
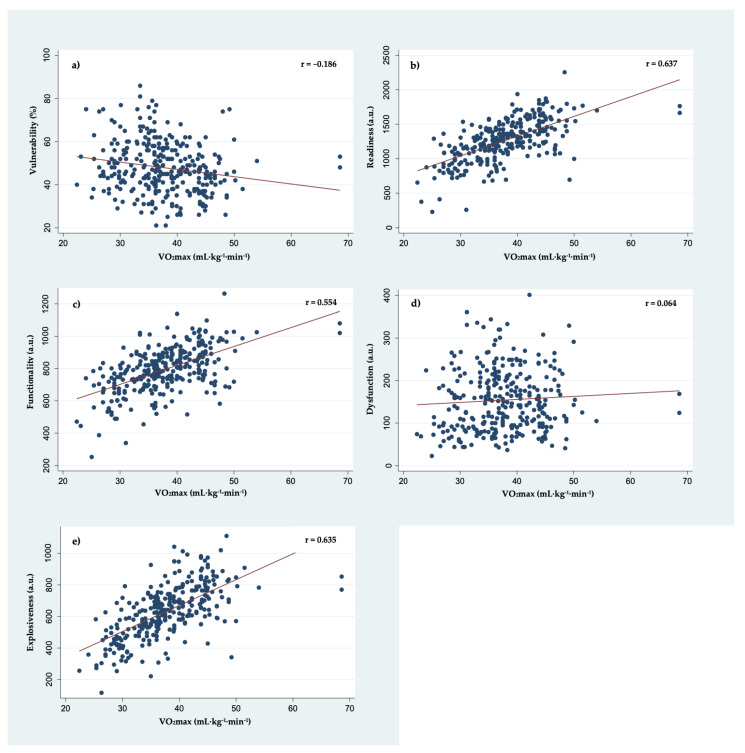
Correlation plots of VO_2_max with (**a**) vulnerability, (**b**) readiness, (**c**) functionality, (**d**) dysfunction, and (**e**) explosiveness scores derived from a 3D-MCS during functional movement screening protocol.

Pearson product–moment correlation coefficients between health-related physical fitness variables and five functional movement analysis scoring scales are presented in Table 3. BF% showed a weak positive correlation with vulnerability (*p* = 0.006), and as expected moderate-to-strong negative correlations with readiness, explosiveness, and functionality scores (all *p* < 0.001). A detailed graphical representation between BF% and five functional movement assessment scoring scales is presented in Figure 1. Similarly, but opposite to BF%, VO_2_max showed a weak negative correlation with vulnerability (*p* = 0.001) and moderate-to-strong positive correlations with readiness, explosiveness, and functionality scores (all *p* < 0.001). A detailed graphical representation between VO_2_max and five functional movement assessment scoring scales is presented in Figure 2. Moreover, HDL-C showed a weak positive correlation with vulnerability scores (*p* = 0.008) and a weak negative correlation with explosiveness scores (*p* = 0.008). In addition, linear regression analysis coefficients for each health-related fitness parameter variable and their respective contributions to the overall prediction model of five functional movement screening scores are presented in Table 4. While multiple findings are of interest, age and sex significantly contributed to the linear regression prediction model for all scoring scales except dysfunction.

## 4. Discussion

As a part of a wide range of health-related physical fitness parameters, previous research has shown that performance on sub-maximal and maximal exercise stress tests and functional movement screenings have been strongly correlated with waist circumference, BMI, BF%, and HDL-C levels [8,21,22,23]. However, there is a notable gap in the scientific literature examining the relationship between some of the key health-related physical fitness parameters and functional movement screenings assessed via innovative 3D-MCS, especially in non-academic and clinical settings. The findings of the present study indicate that the algorithm-derived functional movement screening scores (i.e., readiness, functionality, explosiveness) obtained from an innovative 3D-MCS have moderate-to-strong correlations with traditional health-related physical fitness parameters such as estimated VO_2_max and BF%. While reaching the level of statistical significance, the vulnerability score showed a weak positive relationship with BF% and weak negative relationship with VO_2_max. In addition, as with the outcomes of traditional fitness screening methodologies, the regression models used for prediction of algorithm-derived scoring scales (i.e., functionality, explosiveness, vulnerability, readiness) were significantly influenced by the age and sex of the participants. Therefore, the results of the present study support the use of a 3D-MCS as a non-invasive testing alternative capable of providing health practitioners with additional information regarding some of the key health-related physical fitness parameters. 

Mechanisms related to human motion, whether biomechanical or biomolecular, are interactive and complex in nature. A considerable amount of scientific literature has documented a broad spectrum of health-related benefits resulting from consistent participation in various types of physical activities [24,25,26,27,28,29]. Due to the dose–response relationship between physical activity and health, it is recommended that every adult should perform a mix of vigorous- and moderate-intensity physical activities targeted towards the maintenance and improvement of muscular strength and endurance [27]. In view of these health-related physical fitness benefits, considerable interest has been directed towards the development of screening tests to assess participants’ current cardiovascular and strength status, and from these results to initiate personalized exercise or fitness prescriptions and compare improvements in performance via repeated post-training testing [8,21,30]. Currently, it is hardly a norm for patients in clinical settings to perform strenuous exercise tests, especially if they already possess certain health-related limitations or contraindications. Due to the non-invasive nature of the assessment, based on the findings of the present study, the use of 3D-MCS may serve as a testing alternative to estimate some of the key health-related fitness parameters (e.g., VO_2_max) based on the five functional movement screening scores. This testing modality also allows for rapid data analysis, and it is not labor-intensive. It requires one or two trained individuals to operate the system and it takes approximately 7–10 min to complete the overall screening protocol, with results being available to a practitioner in approximately 30 s. Moreover, when used in the adult population, the confidence in the test measurement validity of 3D-MCS for assessment of functional movement parameters has been successfully investigated [13,14,15,16,17].

An additional advantage of a 3D-MCS relates to the objective-centered results obtained from multiple body motions (i.e., upper- and lower-body). These joint and motion-specific quantitative results may provide important and pertinent information for participant-specific exercise prescription and assure optimal long-term improvements in some of the key health-related physical fitness performance parameters. Further, an assessment of how effective compliance to an exercise prescription has been for a participant may be made by conducting post-exercise screenings and comparing the quantitative pre-minus-post results. While the traditional functional movement screening protocol has been and continues to be a meaningful health-related physical fitness assessment tool, assigning various outcome measures for this test without relying on objective-centered results provided by innovative 3D-MCS could potentially induce a certain degree of measurement error when examining pre- to post-exercise intervention results. 

As previously noted, algorithm-derived functional movement screening scales (i.e., readiness, functionality, explosiveness) showed moderate-to-strong correlations with measurements of BF% and estimated VO_2_max. In the future, perhaps these or similarly derived scoring scales could be used to either assess or further classify the status of an individual’s body composition or cardiovascular fitness compared to their age- and sex-based peers. In addition to potentially using 3D-MCS as a measure of traditional health-related physical fitness parameters (i.e., body composition and cardiovascular fitness), further analysis of upper- and lower-body movements (Table 1) may provide additional information necessary for the development of individually tailored exercise training protocols. For example, improvements in certain joint-related weaknesses or strength imbalances may be effectively targeted as part of the overall exercise prescription regimen. Assessment of the effectiveness of these movement-specific recommendations in relation to an individual’s fitness training or relative to changes in body composition or fitness (i.e., VO_2_max), as assessed by algorithm-derived scoring scales, may serve as an additional application of 3D-MCS. 

While the findings of the present study were obtained from a large sample size from participants who underwent clinically supervised health-related fitness testing protocols, this study is not without limitations. The inclusion criterion was solely based on examining apparently healthy individuals. Therefore, this may limit the applicability of the findings to populations with clinically significant joint injuries or other related disabilities. Further, the mean age of the population examined in the present study was 50.9 ± 10.3 years. Therefore, how consistently the correlations and linear-regression-related equations will relate to a much younger or much older population is uncertain and should be addressed in follow-up investigations.

## 5. Conclusions

The findings of the present study indicate that the algorithm-derived functional movement scoring scales (i.e., readiness, functionality, explosiveness) obtained from an innovative 3D-MCS showed moderate-to-strong correlations with traditional health-related physical fitness parameters such as estimated VO_2_max and BF%. While reaching the level of statistical significance, the vulnerability score showed a weak positive correlation with BF% and weak negative correlation with VO_2_max. Although previous research has demonstrated agreement with other motion assessment technologies, further work is warranted to more thoroughly determine biological and technological variability associated with this assessment modality. However, it appears that 3D-MCS may be used as a non-invasive testing alternative or in conjunction with currently implemented traditional testing modalities to provide health practitioners with additional information regarding some of the key health-related physical fitness parameters, especially within non-academic environments such as wellness and clinical settings. 

## Figures and Tables

**Table 1:** List and description of 19 body movements incorporated into the functional movement screening protocol.

Specific Body Movement	Description of Movement
Shoulder Abduction and Adduction	Start with arms out in a T-position, then raise arms overhead and lower hands to sides.
Shoulder Horizontal Abduction	Start with arms straight out in front of body, separating arms, reach back behind body, and return to the starting position.
Shoulder Internal and External Rotation	Start with arms in a goalpost position, holding this position, rotate arms upward and back, followed by rotating arms forward and down.
Shoulder Flexion and Extension	Start with arms to side and with palms facing inward, raise arms upward as far as possible and then back as far as possible.
Trunk Rotation	Start with arms upward and in a goalpost position, the rotate as far as possible to the left and then as far as possible to the right, keeping hips in a forward position.
Bilateral Squat	Start with feet forward and shoulder distance apart, and while holding a light bar directly above head, lower body downward as far as possible.
Right Leg (Unilateral) Squat	Start by raising left foot off the ground and while balancing on the right leg, lower body down as far as possible on the standing leg and return to the starting position.
Left Leg (Unilateral) Squat	Start by raising right foot off the ground and while balancing on the left leg, lower body down as far as possible on the standing leg and return to the starting position.
Right Leg Lunge	Start with body and feet in a forward position, then take a big step forward with right foot only and lower body toward the ground. Return to the starting position.
Left Leg Lunge	Start with body and feet in a forward position, then take a big step forward with left foot only and lower body toward the ground. Return to the starting position.
Right Leg Balance	Start with body and feet in a forward position, then raise the left slightly off the ground. Standing on the right leg, balance body for 30 s. Hopping on one leg is allowed as long as left foot does not touch the ground.
Left Leg Balance	Start with body and feet in a forward position, then raise the right leg slightly off the ground. Standing on the left leg, balance body for 30 s. Hopping on one leg is allowed as long as right foot does not touch the ground.
Bilateral Standing Vertical Jump	Start with feet forward, legs straight and arms extended backwards as far as possible, then jump as high as possible off both legs.
Right Unilateral Jump	Start with feet forward, legs straight and arms extended backwards as far as possible, raise left leg off ground then jump as high as possible off right leg.
Left Unilateral Jump	Start with feet forward, legs straight and arms extended backwards as far as possible, raise right leg off ground then jump as high as possible off left leg.
Concentric Jump	Start with feet forward, legs bent to a near 90 degree angle and arms extended backwards as far as possible, then jump as high as possible off both legs.
Five Hops Right Leg	Start with feet forward, lift left leg to a near 90 degree angle, then jump as high as possible off right leg, five consecutive times.
Five Hops Left Leg	Start with feet forward, right left leg to a near 90 degree angle, then jump as high as possible off left leg, five consecutive times.
Depth Jump	Begin standing on the box. With either foot, step off the box landing on two feet. Immediately jump for height, spending as little time on the ground as possible. Arms may be used for upward momentum.

**Table 2:** Demographic data and health-related physical fitness test results ($\bar{x}$ ± SD).

Variables	All Participants	Male	Female	*p*-Value
Age (years)	50.9 ± 10.3	50.9 ± 10.3	50.9 ± 10.6	0.990
Height (cm)	177.3 ± 9.4	181.9 ± 6.4	166.1 ± 6.4	<0.001
Weight (kg)	84.8 ± 17.8	90.6 ± 15.1	70.4 ± 15.5	<0.001
Waist Circumference (cm)	95.5 ± 13.2	98.6 ± 11.7	88.1 ± 14.2	<0.001
Hip Circumference (cm)	103.4 ± 8.9	103.6 ± 7.6	102.9 ± 11.2	0.430
Body Mass Index (kg·m^2^)	26.9 ± 4.5	27.4 ± 4.0	25.5 ± 5.4	<0.001
Body Fat (%)	28.8 ± 8.1	26.7 ± 7.3	34.0 ± 7.8	<0.001
VO_2_max (mL·kg^−1^·min^−1^)	37.7 ± 6.6	39.6 ± 6.1	32.7 ± 4.8	<0.001
Cardiorespiratory Fitness (%)	66.8 ± 20.2	68.7 ± 20.5	66.1 ± 20.1	0.310
HDL-cholesterol (mg·dL^−1^)	50.7 ± 13.1	47.9 ± 11.0	57.8 ± 15.1	<0.001
Readiness (a.u.)	1257.5 ± 299.5	1332.5 ± 274.3	1070.8 ± 278.7	<0.001
Explosiveness (a.u.)	631.1 ± 169.5	685.7 ± 145.0	494.0 ± 148.2	<0.001
Functionality (a.u.)	790.5 ± 139.8	816.8 ± 130.1	724.8 ± 142.1	<0.001
Dysfunction (a.u.)	153.8 ± 72.0	160.7 ± 68.8	136.7 ± 77.3	0.010
Vulnerability (%)	47.9 ± 12.0	46.7 ± 11.2	51.1 ± 13.2	<0.001

**Table 3:** Pearson product–moment correlation coefficients (*r*) between health-related fitness parameters and five functional movement screening scores (i.e., readiness, explosiveness, functionality, dysfunction, and vulnerability) and their respective levels of statistical significance.

	Vulnerability		Readiness	
Variable	Coefficient	*p*-Value	Coefficient	*p*-Value
Body Mass Index (kg·m^−2^)	−0.044	0.448	−0.255	<0.001
Waist Circumference (cm)	−0.030	0.602	−0.255	<0.001
Hip Circumference (cm)	−0.038	0.506	−0.276	<0.001
Body Fat (%)	0.158	0.006	−0.593	<0.001
VO_2_max (mL·kg^−1^·min^−1^)	−0.186	0.001	0.637	<0.001
Cardiorespiratory Fitness (%)	0.069	0.235	0.273	<0.001
HDL-cholesterol (mg·dL^−1^)	0.151	0.008	−0.098	0.086
	**Functionality**		**Dysfunction**	
**Variable**	**Coefficient**	***p*-Value**	**Coefficient**	***p*-Value**
Body Mass Index (kg·m^−2^)	−0.311	<0.001	0.059	0.300
Waist Circumference (cm)	−0.308	<0.001	0.092	0.110
Hip Circumference (cm)	−0.316	<0.001	0.020	0.725
Body Fat (%)	−0.522	<0.001	−0.006	0.915
VO_2_max (mL·kg^−1^·min^−1^)	0.554	<0.001	0.064	0.269
Cardiorespiratory Fitness (%)	0.333	<0.001	0.069	0.235
HDL-cholesterol (mg·dL^−1^)	0.016	0.788	−0.029	0.614
	**Explosiveness**			
**Variable**	**Coefficient**	***p*-Value**		
Body Mass Index (kg·m^−2^)	−0.124	0.031		
Waist Circumference (cm)	−0.078	0.178		
Hip Circumference (cm)	−0.171	0.003		
Body Fat (%)	−0.569	<0.001		
VO_2_max (mL·kg^−1^·min^−1^)	0.635	<0.001		
Cardiorespiratory Fitness (%)	0.177	0.002		
HDL-cholesterol (mg·dL^−1^)	−0.152	0.008		

**Table 4:** Unstandardized beta coefficients for the linear regression model used for the prediction of five functional movement screening scores (i.e., readiness, explosiveness, functionality, dysfunction, and vulnerability) based on health-related fitness parameters examined in the present study (*p*-value = represents statistically significant contribution of each predictor variable; sex = 0 for female, 1 for male; 95% CI = 95% confidence interval).

	Vulnerability			Readiness		
Variable	Coefficient	*p*-Value	95% CI	Coefficient	*p*-Value	95% CI
Sex	−1.076	0.043	−7.31, 5.16	214.067	<0.001	105.95, 322.18
Age at Testing (years)	0.299	0.010	0.07, 0.52	−13.499	<0.001	−17.41, −9.59
Body Mass Index (kg·m^−2^)	−0.129	0.757	−0.95, 0.69	0.217	0.976	−13.96, 14.40
Waist Circumference (cm)	−0.091	0.806	−0.82, 0.64	−5.860	0.362	−18.49, 6.77
Hip Circumference (cm)	−0.184	0.645	−0.97, 0.60	−3.456	0.618	−17.09, 10.18
Body Fat (%)	0.293	0.073	−0.03, 0.61	−7.846	0.006	−13.40, −2.30
VO_2_max (mL·kg^−1^·min^−1^)	0.025	0.928	−0.53, 0.58	−1.533	0.754	−11.15, 8.09
Cardiorespiratory Fitness (%)	0.019	0.808	−0.13, 0.17	3.371	0.012	0.76, 5.60
HDL-cholesterol (mg·dL^−1^)	0.052	0.409	−0.07, 0.17	−2.536	0.020	−4.67, −0.40
Constant	34.531	0.035	2.54, 66.53	2337.686	<0.001	1783.01, 2892.36
	**Functionality**			**Dysfunction**		
**Variable**	**Coefficient**	***p*-Value**	**95% CI**	**Coefficient**	***p*-Value**	**95% CI**
Sex	122.75	<0.001	63.86, 1181.65	38.090	0.055	−0.89, 77.07
Age at Testing (years)	−5.943	<0.001	−8.07, −3.81	0.178	0.803	−1.23, 1.59
Body Mass Index (kg·m^−2^)	−2.285	0.561	−10.01, 5.44	1.236	0.635	−3.88, 6.35
Waist Circumference (cm)	−3.572	0.308	−10.45, 3.31	0.228	0.922	−4.33, 4.78
Hip Circumference (cm)	−1.801	0.634	−9.23, 5.63	−1.302	0.603	−6.22, 3.62
Body Fat (%)	−0.904	0.557	−3.93, 2.12	1.523	0.135	−0.48, 3.52
VO_2_max (mL·kg^−1^·min^−1^)	−2.772	0.299	−8.01, 2.47	−0.293	0.868	−3.76, 3.18
Cardiorespiratory Fitness (%)	2.400	0.001	0.98, 3.82	0.727	0.130	−0.21, 1.67
HDL-cholesterol (mg·dL^−1^)	−0.484	0.413	−1.65, 0.68	0.116	0.768	−0.65, 0.88
Constant	1269.82	<0.001	967.68, 1571.97	41.851	0.681	−158.11, 241.81
	**Explosiveness**					
**Variable**	**Coefficient**	***p*-Value**	**95% CI**			
Sex	118.400	<0.001	57.56, 179.24			
Age at Testing (years)	−7.003	<0.001	−9.20, −4.80			
Body Mass Index (kg·m^−2^)	4.140	0.312	−3.91, 12.19			
Waist Circumference (cm)	2.310	0.530	−4.92, 9.54			
Hip Circumference (cm)	−4.291	0.273	−11.98, 3.39			
Body Fat (%)	−6.593	<0.001	−9.72, −3.47			
VO_2_max (mL·kg^−1^·min^−1^)	0.879	0.748	−4.51, 6.27			
Cardiorespiratory Fitness (%)	1.305	0.080	−0.16, 2.77			
HDL-cholesterol (mg·dL^−1^)	−0.773	0.218	−2.00, 0.46			
Constant	987.354	<0.001	672.01, 1302.70			

## Data Availability

The data are not publicly available due to IRB-imposed restrictions.

## References

[1] Riebe D., Ehrman J.K., Liguori G., Magal M. (2018). ACSM’s Guidelines for Exercise Testing and Prescription.

[2] President’s Council of Physical Fitness and Sports Definitions—Health, Fitness, and Physical Activity. http://purl.access.gpo.gov/GPO/LPS21074.

[3] Gordon D.J., Probstfield J.L., Garrison R.J., Neaton J.D., Castelli W.P., Knoke J.D., Jacobs D.R., Bangdiwala S., Tyroler H.A. (1989). High-density lipoprotein cholesterol and cardiovascular disease. Four prospective American studies. Circulation.

[4] Assmann G., Schulte H., von Eckardstein A., Huang Y. (1996). High-density lipoprotein cholesterol as a predictor of coronary heart disease risk: The PROCAM experience and pathophysiological implications for reverse cholesterol transport. Atherosclerosis.

[5] Chapman M.J., Ginsberg H.N., Amarenco P., Andreotti F., Boren J., Catapano A.L., Descamps O.S., Fisher E., Kovanen P.T., Kuivenhoven J.A. (2011). Triglyceride-rich lipoproteins and high-density lipoprotein cholesterol in patients at high risk of cardiovascular disease: Evidence and guidance for management. Eur. Heart J..

[6] Cook G., Burton L., Hoogenboom B. (2006). Pre-participation screening: The use of fundamental movements as an assessment of function—Part 1. N. Am. J. Sports Phys. Ther..

[7] Cook G., Burton L., Hoogenboom B. (2006). Pre-participation screening: The use of fundamental movements as an assessment of function—Part 2. N. Am. J. Sports Phys. Ther..

[8] Farrell S.W., Pavlovic A., Barlow C.E., Leonard D., DeFina J.R., Willis B.L., DeFina L.F., Haskell W.L. (2021). Functional movement screening performance and association with key health markers in older adults. J. Strength Cond. Res..

[9] Chorba R.S., Chorba D.J., Bouillon L.E., Overmyer C.A., Landis J.A. (2010). Use of a functional movement screening tool to determine injury risk in female collegiate athletes. N. Am. J. Sports Phys. Ther..

[10] Kiesel K., Plisky P.J., Voight M.L. (2007). Can serious injury in professional football be predicted by a preseason functional movement screen?. N. Am. J. Sports Phys. Ther..

[11] Lisman P., O’Connor F.G., Deuster P.A., Knapik J.J. (2012). Functional movement screen and aerobic fitness predict injuries in military training. Med. Sci. Sports Exerc..

[12] Garrison M., Westrick R., Johnson M.R., Benenson J. (2015). Association between the functional movement screen and injury development in college athletes. Int J. Sports Phys. Ther..

[13] Corazza S., Mundermann L., Chaudhari A.M., Demattio T., Cobelli C., Andriacchi T.P. (2006). A markerless motion capture system to study musculoskeletal biomechanics: Visual hull and simulated annealing approach. Ann. Biomed. Eng..

[14] Cabarkapa D., Fry A.C., Mosier E.M., Validity of 3-D markerless motion capture system for assessing basketball dunk kinetics—A case study. *Sport J.* (2020). http://thesportjournal.org/article/tag/basketball/.

[15] Harsted S., Holsgaard-Larsen A., Hestbaek L., Boyle E., Lauridsen H.H. (2019). Concurrent validity of lower extremity kinematics and jump characteristics captured in pre-school children by a markerless 3D motion capture system. Chiropr. Man. Ther..

[16] Perrott M.A., Pizzari T., Cook J., McClelland J.A. (2017). Comparison of lower limb and trunk kinematics between markerless and marker-based motion capture systems. Gait Posture.

[17] Sandau M., Koblauch H., Moeslund T.B., Aanaes H., Alkjaer T., Simonsen E.B. (2014). Markerless motion capture can provide reliable 3D gait kinematics in the sagittal and frontal plane. Med. Eng. Phys..

[18] Bruce R.A., Kusumi F., Hosmer D. (1973). Maximal oxygen intake and nomographic assessment of functional aerobic impairment in cardiovascular disease. Am. Heart J..

[19] Pollock M.L., Bohannon R.L., Cooper K.H., Ayres J.J., Ward A., White S.R., Linnerud A.C. (1976). A comparative analysis of four protocols for maximal treadmill stress testing. Am. Heart J..

[20] Pollock M.L., Foster C., Schmidt D., Hellman C., Linnerud A.C., Ward A. (1982). Comparative analysis of physiologic responses to three different maximal graded exercise test protocols in healthy women. Am. Heart J..

[21] Lollgen H., Leyk D. (2018). Exercise testing in sports medicine. Dtsch. Arztebl. Int..

[22] Scudamore E.M., Stevens S.L., Fuller D.K., Coons J.M., Morgan D.W. (2019). Functional movement screen items predict dynamic balance under military torso load. Mil. Med..

[23] Trinidad-Fernandez M., Gonzalez-Sanchez M., Cuesta-Vargas A.I. (2019). Is a low functional movement screen score (≤14/21) associated with injuries in sport? A systematic review and metaanalysis. BMJ Open Sport Exerc. Med..

[24] Barnett A., Smith B., Lord S.R., Williams M., Baumand A. (2003). Community-based group exercise improves balance and reduces falls in at-risk older people: A randomized controlled trial. Age Ageing.

[25] Blair S.N., Kohl H.W., Paffenbarger R.S., Clark D.G., Cooper K.H., Gibbons L.W. (1989). Physical fitness and all-cause mortality. A prospective study of healthy men and women. JAMA.

[26] Lee I.M., Paffenbarger R.S., Hsieh C. (1991). Physical activity and risk of developing colorectal cancer among college alumni. J. Nat. Cancer Inst..

[27] Leon A.S., Connett J., Jacobs D.R., Rauramaa R. (1987). Leisure-time physical activity levels and risk of coronary heart disease and death. The multiple risk factor intervention trial. JAMA.

[28] Taylor C.B., Sallis J.F., Needle R. (1985). The relation of physical activity and exercise to mental health. Public Health Rep..

[29] Haskell W.L., Lee I.M., Pate R.R., Powell K.E., Blair S.N., Franklin B.A., Macera C.A., Heath G.W., Thompson P.D., Bauman A. (2007). Physical activity and public health: Updated recommendation for adults from the American College of Sports Medicine and the American Heart Association. Circulation.

[30] Voet N.B., van der Kooi E.L., Riphagen I.I., Lindeman E., van Engelen B.G., Geurts A.C. (2019). Strength training and aerobic exercise training for muscle disease. Cochrane Database Syst. Rev..

